# Cannabinoid type 2 receptors inhibit GABA_A_ receptor-mediated currents in cerebellar Purkinje cells of juvenile mice

**DOI:** 10.1371/journal.pone.0233020

**Published:** 2020-05-21

**Authors:** Sriity Melley Sadanandan, Tabita Kreko-Pierce, Shailesh N. Khatri, Jason R. Pugh

**Affiliations:** 1 Department of Cellular and Integrative Physiology, University of Texas Health Science Center at San Antonio, San Antonio, TX, United States of America; 2 Center for Biomedical Neuroscience, University of Texas Health Science Center at San Antonio, San Antonio, Texas, United States of America; Medical College of Wisconsin, UNITED STATES

## Abstract

Signaling through the endocannabinoid system is critical to proper functioning of the cerebellar circuit. However, most studies have focused on signaling through cannabinoid type 1 (CB1) receptors, while relatively little is known about signaling through type 2 (CB2) receptors. We show that functional CB2 receptors are expressed in Purkinje cells using a combination of immunohistochemistry and patch-clamp electrophysiology in juvenile mice. Pharmacological activation of CB2 receptors significantly reduces inhibitory synaptic responses and currents mediated by photolytic uncaging of RuBi-GABA in Purkinje cells. CB2 receptor activation does not change the paired-pulse ratio of inhibitory responses and its effects are blocked by inclusion of GDP-β-S in the internal solution, indicating a postsynaptic mechanism of action. However, CB2 receptors do not contribute to depolarization induced suppression of inhibition (DSI), indicating they are not activated by endocannabinoids synthesized and released from Purkinje cells using this protocol. This work demonstrates that CB2 receptors inhibit postsynaptic GABA_A_ receptors by a postsynaptic mechanism in Purkinje cells. This represents a novel mechanism by which CB2 receptors may modulate neuronal and circuit function in the central nervous system.

## Introduction

Many studies have demonstrated that synaptic transmission and plasticity in the cerebellar circuit depends on proper functioning of the endocannabinoid system [1]. Most studies have focused on the roles of cannabinoid type1 (CB1) receptors. In the cerebellum, CB1 receptors are primarily expressed in the presynaptic terminals of granule cells, molecular layer interneurons, and climbing fibers, all of which synapses onto Purkinje cells [2–4]. Following depolarization, Purkinje cells synthesize and release endocannabinoids, which travel retrogradely to activate presynaptic CB1 receptors and inhibit transmitter release [5–8]. CB1 receptor activity is also required for long-term plasticity at parallel fiber-Purkinje cells synapses [9–11], widely thought to be a critical site of plasticity for cerebellar learning [12–14]. In contrast, the expression and function of cannabinoid type2 (CB2) receptors in the cerebellum has received comparatively little attention.

CB2 receptors have been considered a peripheral receptor due to high expression outside the central nervous system (CNS), primarily in the immune system [15]. However, an increasing number of studies have also begun to observe CB2 receptor expression in the CNS, including the cerebellum, raising the possibility that CB2 receptors modulate neuronal or synaptic function. In Purkinje cells, CB2 receptor mRNA [16–18] and protein [17, 19, 20, but see 21] expression have been observed. Furthermore, post-mortem studies of human patients with spinocerebllear ataxia show an increase in CB2 receptor expression in Purkinje cells [20], suggesting CB2 receptors contribute to proper signaling in the cerebellar circuit. However, functional investigations of CB2 receptors in Purkinje cells have not been reported.

In order to more fully understand expression and function of CB2 receptors in cerebellar Purkinje cells, we have investigated these receptors using a combination of immunohistochemistry and whole-cell patch clamp electrophysiology. We find that activation of CB2 receptors with specific agonists inhibits postsynaptic GABA_A_ receptor-mediated currents. This reveals a novel mechanism by which cannabinoids may regulate cell excitability and circuit function. However, stimulation of endocannabinoid synthesis and release from Purkinje cells using standard protocols was not sufficient to activate postsynaptic CB2 receptors, suggesting the receptors may only be activated following coordinated endocannabinoid mobilization from multiple Purkinje cells or during exposure to exogenous cannabinoids such as Δ^9^THC.

## Methods

### Animals

All experimental procedures involving animals were approved by the Institutional Animal Care and Use Committee at UT Health San Antonio and followed the guidelines of the *National Institutes of Health's Guide for the Care and Use of Laboratory Animals*. Male and female C57BL/6 mice (Charles River, Wilmington, MA, USA) or CB2R knock-out mice (Jackson Labs, Bar Harbor, ME, USA; line 005786) 14–21 days old were used for all experiments. Animals were kept on a 12/12 hour light dark cycle with *ad libitum* access to food and water.

### Slice preparation

Acute parasagittal brain slices were prepared from the cerebella of male and female C57BL/6 mice as described previously [22]. Mice were deeply anaesthetized with isoflurane before rapid dissection of the cerebellum in accordance with the University of Texas Health Science Centre San Antonio protocols and guidelines. The cerebellum was immediately placed in ice-cold oxygenated (95%O2, 5%CO2) artificial cerebrospinal fluid (aCSF) containing (in mM): 119 NaCl, 26.2 NaHCO3, 2.5 KCl, 1.0 NaH2PO4, 11 glucose, 2 CaCl2, 1.3 MgCl2. Slices (200–300 μm) were cut from the vermis of the cerebellum using a vibratome (Leica Biosystems, Buffalo Grove, IL, USA) and then incubated at 34°C for 30 min after which slices were maintained at room temperature.

### Immunohistochemistry

Parasagittal sections (200 μm) were cut as described above and incubated in 4% PFA at 4°C overnight to fix the tissue. Slices were then incubated overnight with rabbit CB2 receptor polyclonal antibody(1:300, Cayman chemicals, Ann Arbor, MI, USA) and mouse anti-calbindin antibody(1:400, Sigma-Aldrich, St. Louis, MO, USA), followed by a 2 hour incubation with Alexa Fluor 488 goat anti-mouse IgG(1:400,Life technologies) and Alexa Fluor 594 goat anti rabbit IgG(1:400,Life technologies) at room temperature. Sections were mounted on slides using Fluoroshield mounting medium (Sigma-Aldrich, St. Louis, MO, USA). Z-stack images were acquired using a confocal (LSM 710, Zeiss, Oberkochen, Germany) microscope. CB2 receptor and calbindin labelling in max projection images were determined by measuring the average pixel intensity in regions of interest in the granule cell layer, Purkinje cell layer, and molecular layer in ImageJ.

### Electrophysiology

Prior to electrophysiological recordings, slices were transferred to a recording chamber and perfused with aCSF at a flow rate of 2 ml/min using a recirculating pump (Cole-Parmer, Vernon Hills, IL, USA). Purkinje cells were visually identified using an axioskop upright microscope (Ziess, Oberkochen, Germany) with IR/DIC optics. Patch pipettes were pulled from borosilicate glass on a Fleming/Brown micropipette puller (Sutter Instruments, Novato, CA, USA) to a tip resistance of 2–4 Mohms. For experiments involving EPSCs, the bath aCSF solution contained 100 μM picrotoxin to block inhibitory responses and pipettes were filled with an internal solution containing the following (in mM): 137 K-gluconate, 4 KCl, 10 HEPES, 4 MgCl2, 5 EGTA, 4 Na-ATP, 0.5 Na-GTP, 2 QX-314 (pH 7.3–7.4, 285–295 mosm). For experiments involving IPSCs or GABA uncaging, the bath aCSF solution contained 10 μM NBQX to block excitatory synaptic responses and pipettes were filled with an internal solution containing the following (in mM): 135 CsCl, 10 HEPES, 4 NA-ATP, 0.5 NA GTP, 5 EGTA, and 2 QX-314 (pH 7.3–7.4, 285–295 mosm). Access resistance was monitored throughout the recording and cells that did not maintain a stable access resistance were not included for further analysis. Electrophysiological currents were recorded with a Multiclamp 700B amplifier (Molecular Devices, Sunnyvale, CA, USA), filtered at 5 kHz and digitized at 50 kHz. Data were collected using pClamp software (Molecular Devices, Sunnyvale, CA,USA). Excitatory and inhibitory synaptic currents were evoked by stimulation in the molecular layer through a patch pipette filled with aCSF.

To measure the effects of CB2 receptor agonists on excitatory or inhibitory postsynaptic responses, whole-cell patch clamp recordings were made from Purkinje cells and pairs of EPSCs or IPSCs (50 ms inter-stimulus interval) were evoked once every 10 seconds for 5 minutes to establish a stable baseline amplitude. Cells that did not display a stable EPSC/IPSC amplitude over this period were not included for further analysis. The CB2 receptor agonist was then applied to the bath solution and after a 5 minute wash-in period, EPSCs/IPSCs were again evoked once every 10 seconds.

For experiments using GABA uncaging in place of synaptic stimulation 60 μM RuBi-GABA (Tocris, Bristol, UK) was included in the recirculating bath solution. GABA was uncaged by a brief (5 ms) illumination from an 470 nm LED light source (CoolLED, Andover, UK) with a 30 second inter-sweep interval to allow for clearance of GABA between sweeps. Uncaging experiments were performed in a darkened room to prevent unwanted uncaging of RuBi-GABA in the recirculating bath solution.

For experiments involving depolarization-induced suppression of inhibition (DSI) IPSCs were stimulated at 0.5 Hz for 10 s to establish a baseline amplitude. Cells were then depolarized to 0 mV for 2 s, after which the 0.5 Hz stimulation resumed for an additional 70 s. The DSI protocol was repeated once every 1 or 2 min (the results from these two conditions were indistinguishable and the data have been grouped together). The internal solution was composed of (in mM): 140 CsCl, 10 Hepes, 0.5 EGTA, 2 MgCl2, 0.16 CaCl2, 2 QX-314, 4 Na-ATP, 0.5 Na-GTP (pH 7.3–7.4, 280–300 mOsm). DSI was measured by comparing the amplitude of the first three IPSCs after Purkinje cell depolarization to the amplitude of baseline IPSCs. Only cells in which the baseline IPSC amplitude and DSI amplitude were stable prior to application of AM251 or SR144528 were included for further analysis (*<*10% change). Once a stable baseline of DSI was recorded for at least 5 minutes the protocol was repeated for 10–15 mins in the presence of AM251 and SR144528.

#### Data analysis

Data was analyzed in IgorPro (Wavemetrics, Lake Oswego, OR) using the Neuromatic toolkit [23] and custom macros. Statistical significance was determined using two-tailed paired Student’s *t*-tests in Excel (Microsoft, Redmond, WA). Statistical values of *P* ≤ 0.05 were considered significant. Stimulus artifacts have been digitally removed.

## Results

**Fig 1:**
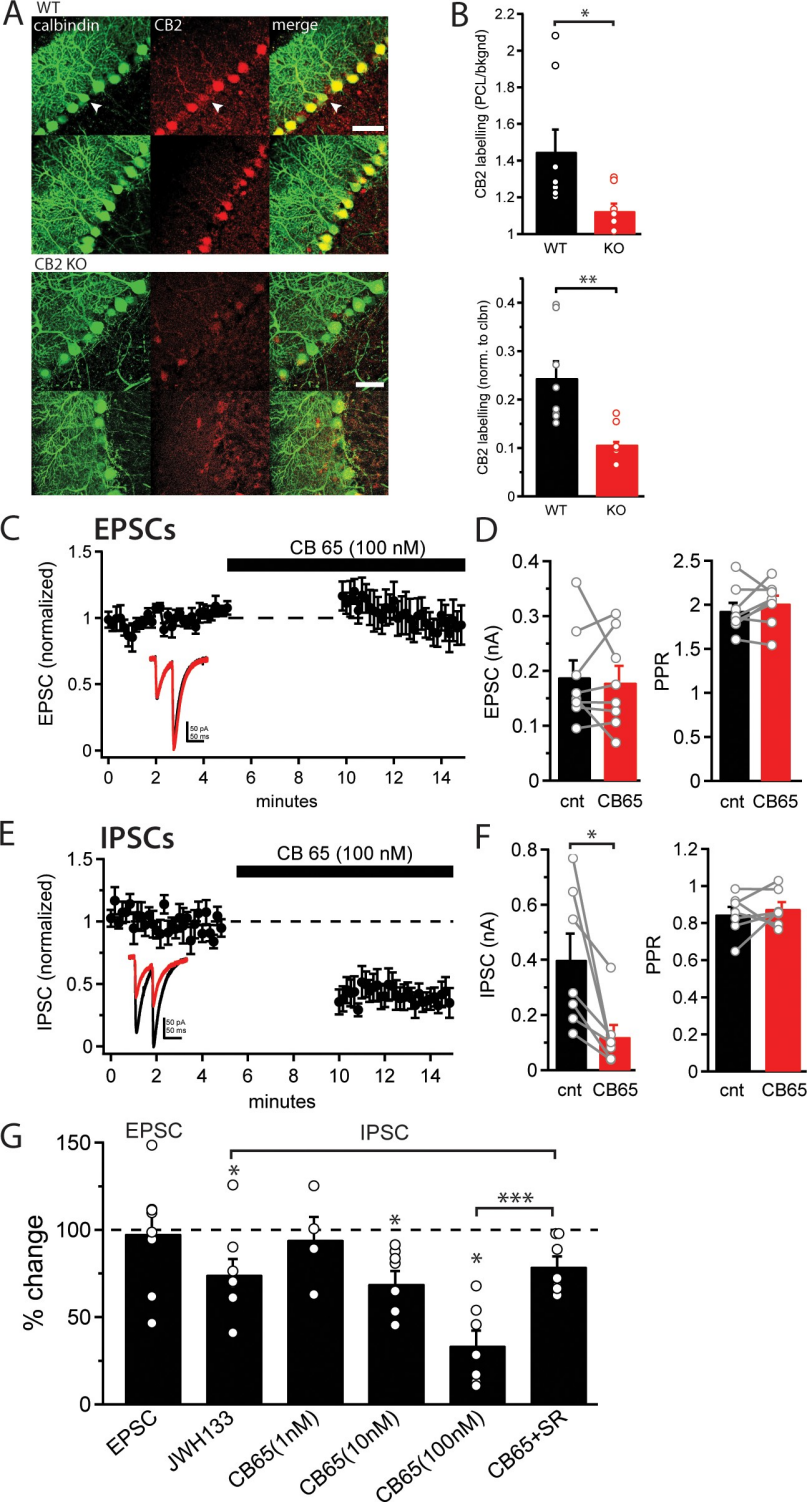
CB2 receptor activation reduces IPSC amplitudes. A) Immunolabeling for calbindin (a Purkinje cell marker, left) and CB2 receptors (middle) in sagittal cerebellar slice from WT (top) and CB2 receptor KO mice (bottom). Arrow indicates Purkinje cell labeled for calbindin but not CB2. B) CB2 receptor labeling in the Purkinje cell layer normalized to background (top) or calbindin labelling (bottom) in tissue from wild type (black) and CB2 receptor KO (red) mice. C) Time-course of average EPSC amplitudes recorded from a Purkinje cell (normalized to the baseline) during bath application of CB 65, a CB2 receptor agonist. Inset: Example EPSCs before (black) and after (red) bath application of CB 65. D) Average EPSC amplitudes (left) and paired-pulse ratio (right) before (black) and after (red) CB 65 application. Data from individual cells plotted as connected gray circles. E) Time-course of average IPSC amplitudes recorded from a Purkinje cell (normalized to the baseline) during bath application of CB 65. Inset: Example IPSCs before (black) and after (red) bath application of CB 65. F) Average IPSC amplitudes (left) and paired-pulse ratio (right) before (black) and after (red) CB 65 application. Data from individual cells plotted as connected gray circles. G) Change in EPSC/IPSC amplitudes (plotted as % of baseline amplitude) following application of JWH133 or CB 65 in control ACSF or in the presence of SR144528 (CB65+SR). Data from individual cells plotted as gray circles. (*) indicates p<0.05, (**) indicates p<0.01 (***) indicates p<0.001.

In order to investigate the expression of CB2 receptors in the cerebellum of juvenile mice we first used immunohistochemistry to label CB2 receptors in cerebellar slices. We found extensive CB2 receptor labeling in the Purkinje cell layer and weak labeling in the granule cell layer, consistent with selective expression of CB2 receptors in Purkinje cells (Fig 1A, top). Interestingly, CB2 receptor expression was mainly limited to Purkinje cell bodies with little expression extending into the dendrites. CB2 receptor expression was often variable, we noted several examples of Purkinje cells with strong calbindin labeling but little or no CB2 receptor labeling (Fig 1A, arrow). CB2 receptor labelling in tissue from CB2 receptor KO mice was significantly reduced (Fig 1A, bottom), with a small amount of labelling still evident in the Purkinje cell layer in some cases, likely due to non-specific labelling by the CB2 receptor antibody. CB2 labelling across genotypes was quantified by measuring the intensity in the Purkinje cell layer normalized to background. We found that labelling in the Purkinje cell layer was 44.7±12.3% greater than background in wild-type tissue and only 12.4±4.2% greater than background in CB2 KO tissue (p = 0.044; n = 8 slices, 3 mice (wt); 7 slices, 2 mice (KO); Fig 1B, top), an approximately 3.5 fold increase in wild-type tissue. Labelling in the granule cell layer was not different between genotypes (p = 0.22), suggesting overall labelling intensity did not differ between genotypes. In order to control for potential differences in labelling efficiency and exposure across slices, we also measured CB2 receptor labelling in Purkinje cell bodies normalized to calbindin labelling. We again found that CB2 receptor labelling in Purkinje cell bodies was significantly greater in wild-type (R/G: 0.24±0.06, n = 8) compared to CB2 KO (R/G: 0.11±0.01, n = 7, p = 0.006; Fig 1B, bottom). While the increased labelling of the Purkinje cell layer suggest CB2 receptors may be expressed in Purkinje cell bodies, the lack of antibody specificity makes it difficult to draw definitive conclusions. We therefore turned to electrophysiological recordings and CB2 receptor pharmacology to determine with greater confidence whether Purkinje cells express functional CB2 receptors.

**Fig 2:**
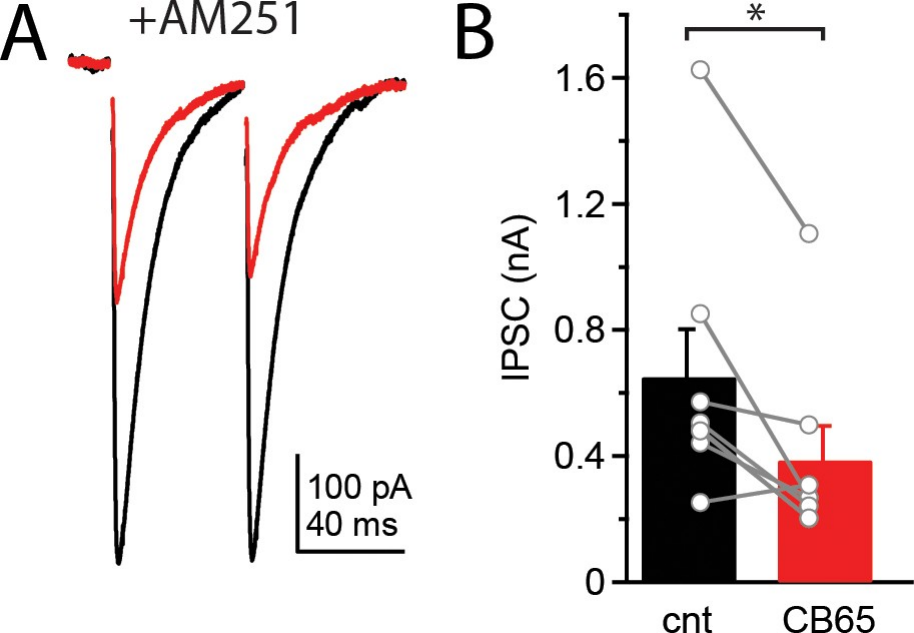
Inhibition of IPSCs is not mediated by CB1 receptors. Example current traces (A) and average amplitudes (B) of evoked IPSCs before (black) and after (red) bath application of CB 65 in the presence of AM251, a CB1 receptor antagonist. Data from individual cells plotted as connected gray circles. (*) indicated p<0.05.

In order to assess whether CB2 receptors are functional and the physiological consequences of their activation, we used whole cell patch clamp recordings from Purkinje cells in acute cerebellar slices. To measure effects on synaptic transmission we recorded evoked excitatory and inhibitory postsynaptic currents (EPSCs and IPSCs) in Purkinje cells before and after application of CB2 receptor agonists. While recording from a Purkinje cell we first evoked EPSCs (inhibitory responses were blocked by 100 μM picrotoxin) once every 10 seconds for 5 minutes to establish a stable baseline of EPSC amplitudes. We then washed on 100 nM CB 65, a selective CB2 receptor agonist [24, 25], for five minutes and again evoked EPSCs. Application of CB 65 had no effect on the amplitude (188.3±30.8 pA versus 179.0±30.1 pA, n = 8, p = 0.72) or paired-pulse ratio (PPR; 1.93±0.09 versus 2.02±0.09, n = 8, p = 0.32) of EPSCs in Purkinje cells (Fig 1C and 1D). However, when the same experiment was performed with evoked IPSCs (excitatory responses were blocked by 10 μM NBQX in the bath), CB 65 substantially reduced the amplitude of IPSCs (100 nM CB 65: 400.3±94.5 pA versus 120.5±43.5 pA, n = 7, p = 0.02; Fig 1E and 1F) in a concentration dependent manner (10 nM CB 65: 493.4±49.2 pA versus 336.2±47.6 pA, n = 7, p = 0.02; Fig 1G). Furthermore, the inhibitory effect of CB 65 was partially blocked by the CB2 receptor competitive antagonist, SR144528 (0.5 μM) (CB 65: 66.2±8.5% inhibition, CB 65+SR144528: 20.9±5.8% inhibition; n = 7; p = 0.0008). However, CB 65 still produced a small, but significant (p = 0.02), depression of IPSC amplitudes in the presence of SR144528, this could result from off-target effects of CB 65 or from CB 65 competing with SR144528 at the ligand binding site. Application of SR144528 alone had no effect on IPSC amplitudes (n = 8, p = 0.99). There was no change in IPSC decay kinetics (tau: 21.7±3.9 ms versus 22.5±4.0, n = 7, p = 0.68) or paired-pulse ratio following CB 65 application (0.85±0.04 versus 0.88±0.04, n = 7, p = 0.54; Fig 1E), suggesting CB2 receptors modulated GABA currents through a postsynaptic mechanism. In order to confirm these results and rule out possible off target effects of CB 65 we repeated these experiments with a second CB2 receptor selective agonist, JWH133 (100 nM). IPSCs were again significantly reduced following application of JWH133 (454.2±45.3pA versus 312.4±9.0 pA, n = 8, p = 0.02; Fig 1G) with no change in the paired-pulse ratio (0.94±0.05 versus 0.88±0.08, n = 8, p = 0.11). It is well established that activation of CB1 receptors causes similar reductions in IPSC amplitude in Purkinje cells through a presynaptic mechanism [7, 26], raising the possibility that CB 65 and JWH133 act through off-target activation of CB1 receptors. In the presence of 2 μM AM251, a CB1 receptor antagonist, application of CB 65 continued to decreased IPSC amplitudes (651.6±150.9pA versus 387.2±107.9pA, n = 8, p = 0.01) with no change in the paired-pulse ratio (0.87±0.02 versus 0.84±0.04, n = 8, p = 0.44; Fig 2), suggesting the inhibition of IPSCs is not mediated by CB1 receptors. These data demonstrate that functional CB2 receptors are expressed in Purkinje cells and their activation significantly reduces IPSC amplitudes with no effect on EPSC amplitudes.

**Fig 3:**
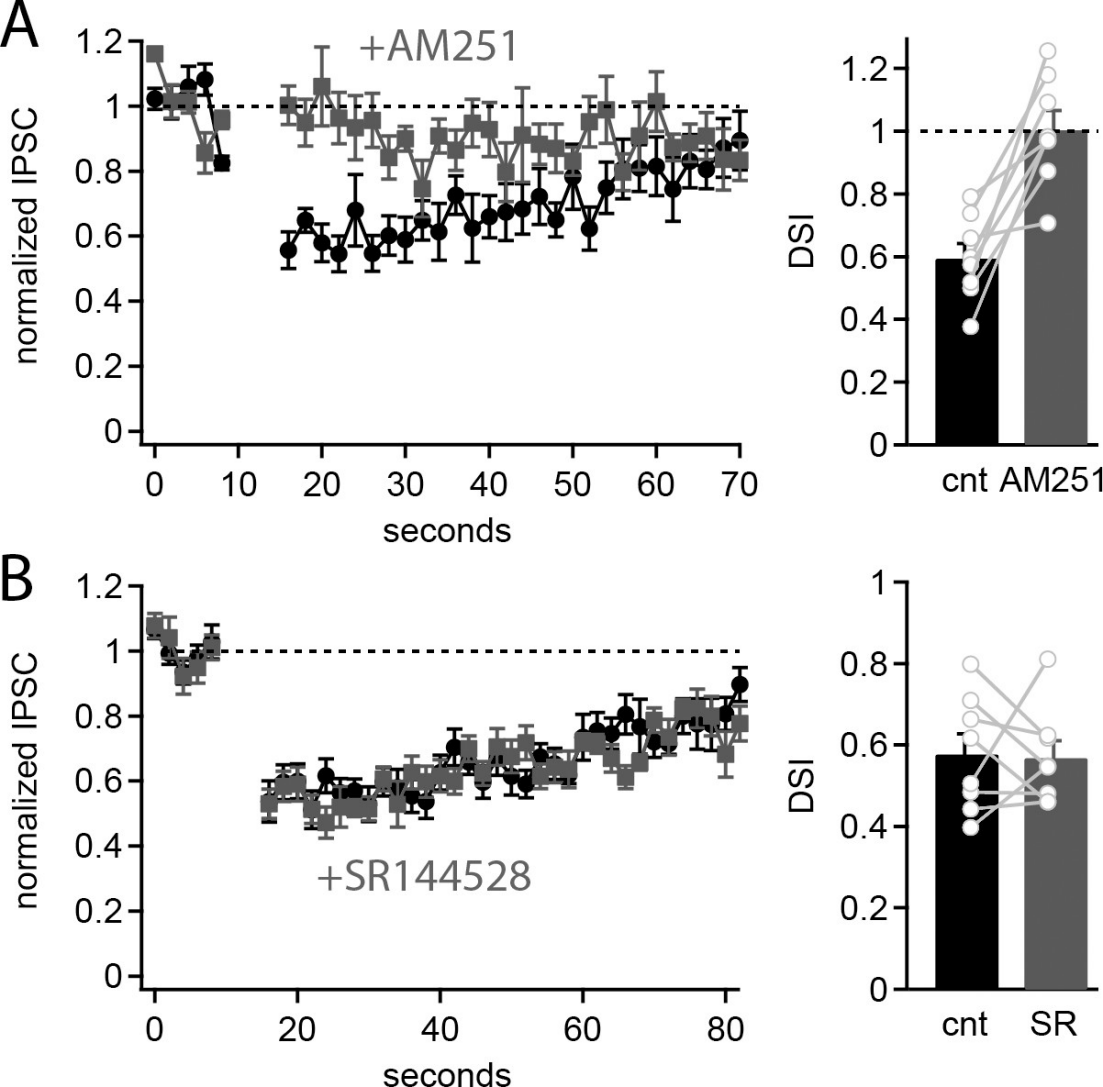
CB2 receptors do not contribute to DSI. A) Average IPSC amplitudes (normalized to the baseline amplitude) over the time-course of the DSI protocol (left) and average inhibition of IPSCs during DSI (right) in control ACSF (black) and in the presence of AM251 (gray). Data from individual cells plotted as connected gray circles. B) Average IPSC amplitudes (normalized to the baseline amplitude) over the time-course of the DSI protocol (left) and average inhibition of IPSCs during DSI (right) in control ACSF (black) and in the presence of SR144528 (gray). Data from individual cells plotted as connected gray circles.

We then investigated whether CB2 receptors are activated by endogenously released cannabinoid compounds. Previous work has shown that strong depolarization of Purkinje cells by either injection of current through a patch pipette or stimulation of excitatory synaptic inputs results in mobilization of endocannabinoids and subsequent activation of presynaptic CB1 receptors [7, 27]. This results in suppression of IPSCs in Purkinje cells lasting tens of seconds referred to as depolarization induced suppression of inhibition (DSI). Because CB2 receptors are also activated by endocannabinoids and result in suppression of IPSCs in Purkinje cells, we investigated whether CB2 receptors also respond to endocannabinoid produced by Purkinje cells and contribute to DSI. To measure DSI, we made whole cell patch clamp recordings from Purkinje cells and stimulated IPSCs at 0.5 Hz. After a 10 second baseline period the Purkinje cell was depolarized to 0 mV for 2 seconds to stimulate endocannabinoid production. Following the depolarization, IPSCs were again evoked at 0.5 Hz for 70–80 seconds. In control ACSF depolarization of the Purkinje cell produced a significant reduction in IPSC amplitude (59.5±4.7% of baseline, n = 8, p = 0.003). However, the suppression of inhibition was completely blocked by application of the CB1 receptor antagonist, AM251 (2 μM; 100.4±6.1% of baseline, n = 8, p = 0.47), suggesting DSI is entirely dependent on CB1 receptors (Fig 3A), and consistent with earlier results [7]. Furthermore, application of a CB2 receptor antagonist, SR144528 (0.5 μM), had no effect on DSI (control ACSF: 57.7±5.0% of baseline, SR144528: 56.9±4.1%of baseline, n = 8; Fig 3B). These data suggest that DSI in Purkinje cells is mediated solely by CB1 receptors with little or no CB2 receptor contribution. This further implies that even though CB2 receptors are expressed in Purkinje cells and can suppress IPSC amplitudes, they are not activated by endogenous cannabinoids synthesized by Purkinje cells during a 2 second depolarization. However, it is possible that other mechanisms of endocannabinoid production in the cerebellum remain unidentified that could activate CB2 receptors.

**Fig 4:**
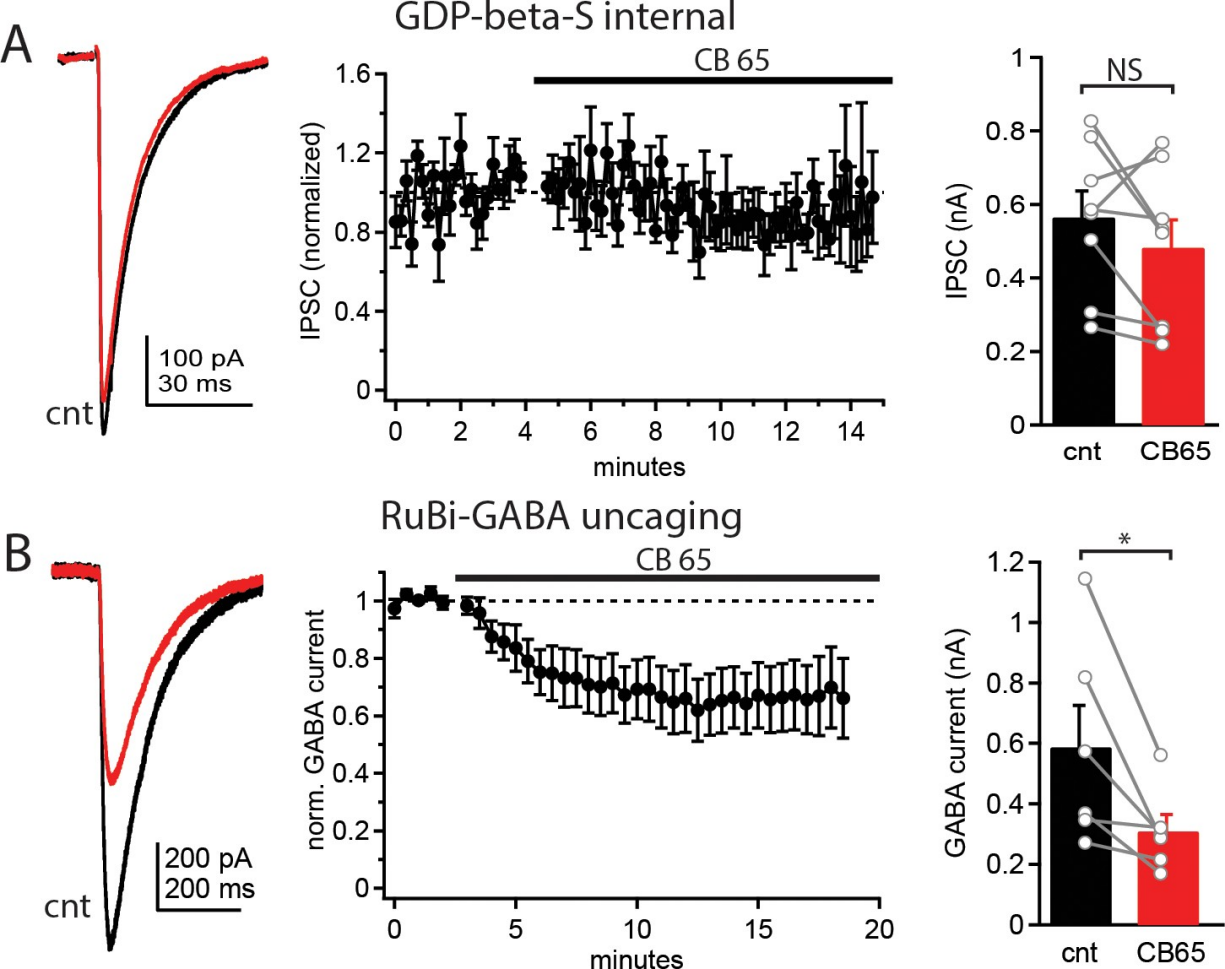
CB2 receptors inhibit postsynaptic GABA_A_ receptors. A) Example current traces of IPSCs (left), average IPSC values over the time-course of the experiment (normalized to the baseline; middle) and average IPSC values (right) before (black) and after (red) CB 65 application when GDP-β-S is included in the internal solution. B) Example current traces following photolytic uncaging of RuBi-GABA (left), average GABA current values over the time-course of the experiment (normalized to the baseline; middle) and average GABA current values (right) before (black) and after (red) CB 65 application. Data from individual cells plotted as connected gray circles. (*) indicated p<0.05.

Previous work provides strong evidence that CB1 receptors in the cerebellum act presynaptically to reduce transmitter release [8]. The localization of CB2 receptors to the Purkinje cell body and lack of effect on the paired-pulse ratio suggest that CB2 receptors act postsynaptically. To determine whether CB2 receptors act pre- or postsynaptically in Purkinje cells we performed two experiments. First, we included GDP-β-S in the internal solution to block G-protein mediated signaling in the patched Purkinje cell. We found that in the presence of GDP-β-S bath application of CB 65 no longer reduced evoked IPSC amplitudes (564.2±71.6pA versus 482.4±75.5pA, n = 8, p = 0.22; Fig 4A), suggesting the effects of CB 65 are dependent on a G-protein coupled receptor in the postsynaptic cell. Second, we replaced stimulation of IPSCs with photolytic uncaging of RuBi-GABA. In these experiments 60 μM RuBi-GABA was included in the bath solution and GABA was uncaged by a brief (5 ms) flash from a 473 nm LED light source. After establishing a stable baseline amplitude of the GABA uncaging current, CB 65 was washed into the bath and uncaging current amplitudes were monitored for an additional ~15 minutes. The uncaging current amplitude was significantly reduced by CB 65 (587.7±138.0pA versus 309.6±55.5pA, n = 6, p = 0.03, Fig 4B) suggesting CB2 receptors directly inhibit postsynaptic GABA_A_ receptors (GABA_B_ receptor-mediated K^+^ currents are blocked by Cs^+^ in the internal solution). These data, together with data showing CB2 receptors are expressed primarily in Purkinje cells (Fig 1A) suggest that CB2 receptors act through a postsynaptic mechanism to inhibit GABA_A_ receptor-mediated currents.

## Discussion

We find that juvenile mice express functional CB2 receptors in Purkinje cells of the cerebellum. Activation of these receptors using specific agonists (CB 65 and JWH133) reduces the amplitude of inhibitory synaptic responses and this effect is partially blocked by a CB2 receptor specific competitive antagonist, SR144528. The incomplete block of CB 65 effects by a CB2 receptor antagonist is likely due to either off-target effects of CB 65 [28] or CB 65 competing off the antagonist at the ligand binding site. However, potential off-target effects of CB 65 would need to also be mediated by postsynaptic G-protein coupled receptors as the inhibitory effects of CB 65 is completely blocked by GDP-β-S in the internal solution (Fig 4A). CB2 receptor activation also inhibits currents evoked by photolytic uncaging of RuBi-GABA on Purkinje cells and the effects of CB2 receptor activation are blocked by inclusion of GDP-β-S in the intracellular solution, suggesting a postsynaptic mechanism of action. These data present a novel mechanism of CB2 receptor action involving inhibition of postsynaptic GABA_A_ receptors. Despite being sensitive to low levels of agonist (10 nM CB 65), Purkinje cell CB2 receptors did not respond to endocannabinoids released from the Purkinje cell in our experiments. Depolarization of Purkinje cells caused robust release of endocannabinoids from the cell as demonstrated by reduction of inhibitory synaptic currents (DSI). However, DSI was blocked by a CB1 receptor antagonist and not affected by a CB2 receptor antagonist, suggesting little or no activation of CB2 receptors by this protocol. While we did not find activation of CB2 receptors by endocannabinoids, it is still likely they play a role in Purkinje cell physiology and may be activated by other protocols not yet tested or during exposure to exogenous cannabinoid compounds. This work focuses on juvenile (2–3 weeks old) mice, an age range at which the major circuit elements and connections of the cerebellum have been established, however, future work will be necessary to determine whether the pattern of CB2 receptor expression or their functional consequences in Purkinje cells is altered with further development.

### CB2 receptor expression in the cerebellum

Several studies have previously described CB2 receptor mRNA and protein expression in the cerebellum [16–21]. However, the pattern of expression found in the cerebellar cortex has varied across studies. We find high expression of CB2 receptor protein in the Purkinje cell layer and relatively weak expression in granule cells in juvenile mice (Fig 1A). This is consistent with the majority of reports which have found high CB2 expression in Purkinje cells [16–17, 19–20]. However, one study observed CB2 receptor expression in the molecular layer, but not Purkinje cells [21] and another found CB2 receptor expression in the vasculature of the cerebellum, but not in any neuronal cell type [29]. Interestingly, our immunolabeling and electrophysiology data both indicate CB2 receptor expression may be variable across Purkinje cells, suggesting CB2 receptor expression may be specific to subsets of Purkinje cells or a plastic property of the cell. In the granule cell layer, one study found high CB2 receptor expression[16] while others found little or no expression in this area [17, 19, 21]. We observed only weak CB2 receptor labelling in the molecular layer which generally co-localized with the dendrites of Purkinje cells, suggesting little CB2 receptor expression in parallel fibers or molecular layer interneurons.

### Functional effects of CB2 receptor activation

Traditionally, CB2 receptors were thought of as peripheral receptors. However, in recent years increasing evidence suggests that CB2 receptors are also widely expressed in neurons of the central nervous system. Despite widespread expression there are relatively few studies of the cellular function of CB2 receptors compared to the more widely studied CB1 receptors. In some neurons, including neurons of the Rostral Ventromedial Medulla, cultured hippocampal neurons, and dopamine neurons of the ventral tegmental area (VTA), CB2 receptors are expressed presynaptically and inhibit transmitter release, seeming to compliment the actions of presynaptic CB1 receptors [30–32]. In CA3 pyramidal neurons of the hippocampus, cortical pyramidal neurons, and VTA dopamine neurons, CB2 receptors have been shown to reduce cell excitability through a variety of postsynaptic mechanisms, including modulation of the Na-bicarbonate co-transporter [33], activation of a chloride current [34, 35], and activation of potassium currents [32, 36, 37]. Finally, chronic activation of CB2 receptors has also been shown to increase excitatory synaptic transmission and spine density in the hippocampus through an ERK-dependent mechanism [38]. As far as we are aware, this work is the first showing direct inhibition of GABA_A_ receptor-mediated currents by CB2 receptors. This is consistent with previous studies which demonstrate modulation of GABA_A_ receptors by other G_i/o_ coupled receptors. GABA_B_ receptors have been shown to enhance tonic GABA_A_ receptor currents in several cell types [39–42], and orexin receptors (which also couple to G_s_ and G_q_ proteins) decrease GABA_A_ receptor currents [43], suggesting regulation of GABA_A_ receptors by signalling cascades linked to G-protein coupled receptors may be a widespread mechanism for controlling neuronal function. These results reveal that CB2 receptor function may be more diverse in central neurons than has been previously appreciated. Future work will be required to determine if other GABAergic neurons also express CB2 receptors and if they inhibit GABA_A_ receptor currents.

### Cannabinoid signaling in cerebellum

While our data present evidence for functional CB2 receptor expression in Purkinje cells, these receptors were not strongly activated by endogenous cannabinoids released from Purkinje cells. Purkinje cells synthesize and release endocannabinoids (primarily 2-AG) in response to depolarization of the membrane potential and subsequent calcium influx [44] and/or following activation of type 1 metabotropic glutamate receptors in dendritic spines [45]. Several studies, and our own data, have shown that release of endocannabinoids from Purkinje cell dendrites is sufficient to activate CB1 receptors expressed in presynaptic terminals and inhibit release of neurotransmitters (Fig 3) [5–6, 46]. Given that CB1 and CB2 receptors both bind 2AG with relatively high affinity [47, 48], it is somewhat surprising that depolarization of Purkinje cells and mobilization of endocannabinoids does not activate CB2 receptors in our experiments. One possible explanation for this discrepancy is that the location of the receptors matters. In Purkinje cells, endocannabinoids are primarily synthesized in the dendrites where they need diffuse only a short distance to bind CB1 receptors on presynaptic terminals. However, our immunolabeling studies suggest that CB2 receptors are primarily expressed at the soma of Purkinje cells (Fig 1A). At parallel fiber synapses, 2-AG release from the Purkinje cell dendrites and activation of presynaptic CB1 receptors appears to be broadly synapses specific [27, 49], suggesting diffusion of 2-AG is relatively restricted in the molecular layer. This is consistent with reports of high MAGL expression, the enzyme primarily responsible for 2-AG degradation, in presynaptic terminals and astrocytes in the molecular layer [49, 50]. This raises the possibility that 2-AG is selectively synthesized in the dendrites of Purkinje cells and is therefore unable to reach CB2 receptors expressed primarily at the soma. If this is the case, one would not expect to observe DSI at basket cell-Purkinje cell synapses at the Purkinje cell soma or axon initial segment. However, in recordings of connected basket cell-Purkinje cell pairs, inhibition of basket cell synaptic responses was observed following Purkinje cell depolarization [51]. This result may be explained by the presence of basket cell synapses onto the dendrites of Purkinje cells in addition to the soma and pinceau region, leaving open the possibility that endocannabinoids are only released from the dendrites.

### Activation by exogenous cannabinoids

While we did not find activation of CB2 receptors in Purkinje cells following release of endogenous cannabinoids, it remains possible that these receptors participate in pathophysiological responses, such exposure to exogenous cannabinoid compounds. CB2 receptor have a relatively high affinity for Δ^9^THC, the primary psychoactive compound in cannabis (K_i_ = 3.1–75.3 nM) [52]. In humans, Δ^9^THC reaches a peak concentration of 400–500 nM in the bloodstream following smoking of a single marijuana joint [53] and concentrations in the brain may be even higher due to the lipophilic nature of these compounds [54]. It is therefore likely that Purkinje cells are exposed to concentrations of Δ^9^THC or other cannabinoid compounds well over the levels necessary to activate CB2 receptors. This suggests that activation of CB2 receptors and inhibition of GABA_A_ receptor-mediated currents may play a role in the physiological effects of cannabinoid use. In particular, CB2 receptor activation is expected to compliment the reduction in inhibitory synaptic transmission mediated by presynaptic CB1 receptors, causing a profound decrease in inhibition in Purkinje cells and potentially resulting in cerebellar dysfunction.

## Supporting information

S1 Data (DOCX)
