# Supplementary material for: Cannabinoid type 2 receptors inhibit GABAA receptor-mediated currents in cerebellar Purkinje cells of juvenile mice
Source: PLoS One. 2020 May 21;15(5):e0233020. doi: 10.1371/journal.pone.0233020 (PMC7241750; doi:10.1371/journal.pone.0233020)
Supplement: S1 Data — (DOCX) [file pone.0233020.s001.docx]

**Cannabinoid type 2 receptors inhibit GABA_A_ receptor-mediated currents in cerebellar Purkinje cells of Juvenile Mice.** Sriity Melley Sadanandan, Tabita Kreko-Pierce, Shailesh N. Khatri and Jason R. Pugh

**Supporting Information:**

**Figure 1:**

**1B:**

**CB2 labelling intensity (average pixel intensity in ROIs from each region)**

|  | **Granule cell layer** | | **PCL/bckgrd** | | **PCL(CB2/calbindin)** | |
| --- | --- | --- | --- | --- | --- | --- |
|  | **WT** | **CB2 KO** | **WT** | **CB2 KO** | **WT** | **CB2 KO** |
|  | 36189 | 37370 | 1.24236 | 1.016441 | 0.151931 | 0.094875 |
|  | 50053 | 47195 | 1.254609 | 1.103432 | 0.17035 | 0.099788 |
|  | 51964 | 48977 | 1.2035 | 1.135235 | 0.180422 | 0.103372 |
|  | 43392 | 60520 | 1.365339 | 1.109699 | 0.396238 | 0.065538 |
|  | 44008 | 52501 | 1.283262 | 1.069285 | 0.225033 | 0.102609 |
|  | 52329 | 34831 | 1.223634 | 1.310023 | 0.166356 | 0.171824 |
|  | 8598 | 47708 | 2.081531 | 1.292811 | 0.271881 | 0.154419 |
|  | 16762 |  | 1.918519 |  | 0.390034 |  |
|  |  |  |  |  |  |  |
| **mean** | 37911.84 | 46899 | 1.45 | 1.12 | 0.244 | 0.106 |
| **std error** | 5869.897 | 3090.035 | 0.12 | 0.04 | 0.035 | 0.014 |
| **t-test** |  | 0.22 |  | 0.044 |  | 0.006 |

**1D:**

|  | **EPSC** |  |  |  |
| --- | --- | --- | --- | --- |
|  | **control** |  | **CB65** |  |
|  | **amplitude** | **PPR** | **amplitude** | **PPR** |
|  | -133.80443 | 1.84751 | -126.5772 | 1.80116 |
|  | -361.436387 | 1.94609 | -223.3612 | 2.35353 |
|  | -159.430667 | 2.17325 | -175.1124 | 2.16992 |
|  | -149.012658 | 1.60335 | -69.28478 | 1.54241 |
|  | -272.117083 | 2.43067 | -304.444 | 2.14298 |
|  | -192.466542 | 1.78134 | -285.899 | 2.04905 |
|  | -95.4015 | 1.86703 | -106.268 | 2.06153 |
|  | -142.938645 | 1.81595 | -141.1209 | 2.01097 |
|  |  |  |  |  |
| **Mean** | -188.325989 | 1.93314875 | -179.0084 | 2.016444 |
| **Std. Error** | 30.77955933 | 0.090888258 | 30.07638 | 0.08736 |
| **n** | 8 | 8 | 8 | 8 |
| **t-test** |  |  | 0.720099 | 0.324917 |

**1F:**

|  | **IPSC** |  |  |  |  |  |
| --- | --- | --- | --- | --- | --- | --- |
|  | **control** | | | **CB65 (100 nM)** | | |
|  | **amplitude** | **PPR** | **tau** | **amplitude** | **PPR** | **tau** |
|  | -280.258 | 0.648087 | 1.543003 | -128.111 | 0.861745 | 1.160436 |
|  | -547.13 | 0.856558 | 1.167463 | -371.582 | 0.792928 | 1.261149 |
|  | -646.745 | 0.920006 | 1.086949 | -82.2886 | 0.764618 | 1.307843 |
|  | -132.709 | 0.786358 | 1.271685 | -37.6859 | 0.850653 | 1.175567 |
|  | -768.295 | 0.9075 | 1.101928 | -81.9793 | 1.02979 | 0.971072 |
|  | -239.581 | 0.825333 | 1.211632 | -40.9928 | 0.857488 | 1.166197 |
|  | -187.093 | 0.985001 | 1.015227 | -101.055 | 0.981236 | 1.019123 |
|  |  |  |  |  |  |  |
| **Mean** | -400.259 | 0.846978 | 1.199698 | -120.528 | 0.876923 | 1.151627 |
| **Std. Error** | 94.4933 | 0.041358 | 0.06556 | 43.5348 | 0.036258 | 0.045622 |
| **n** | 7 | 7 | 7 | 7 | 7 | 7 |
| **t-test** |  |  |  | 0.022318 | 0.536575 | 0.52777 |

**1G:**

|  | **IPSC (pA)** | | | |
| --- | --- | --- | --- | --- |
|  | **control** | **CB65(10nM)** | **control** | **CB65(1nM)** |
|  | -641.614 | -295.244 | -274.841 | -276.422 |
|  | -357.022 | -288.832 | -377.68 | -336.643 |
|  | -542.884 | -246.467 | -846.394 | -1060.55 |
|  | -306.191 | -268.731 | -817.658 | -515.264 |
|  | -462.697 | -246.847 |  |  |
|  | -499.275 | -417.675 |  |  |
|  | -644.439 | -589.452 |  |  |
|  |  |  |  |  |
| **Mean** | -493.446 | -336.178 | -579.143 | -547.219 |
| **Std. Error** | 49.2448 | 47.63057 | 147.6198 | 178.4661 |
| **n** | 7 | 7 | 4 | 4 |
| **t-test** |  | 0.016992 |  | 0.783015 |

|  | **IPSC (SR144528)** | |
| --- | --- | --- |
|  | **control(SR)** | **CB65** |
|  | -681.431 | -666.915 |
|  | -888.499 | -600.215 |
|  | -657.043 | -475.305 |
|  | -218.626 | -213.978 |
|  | -685.802 | -429.502 |
|  | -649.415 | -577.025 |
|  | -640.852 | -425.187 |
|  |  |  |
| **Mean** | -631.667 | -484.018 |
| **Std. Error** | 76.06629 | 56.70183 |
| **n** | 7 | 7 |
| **t-test** |  | 0.015271 |

|  | **IPSC** | | | |
| --- | --- | --- | --- | --- |
|  | **control** | | **JWH133** | |
|  | **amplitude** | **PPR** | **amplitude** | **PPR** |
|  | 567.283 | 0.730169 | 344.667 | 0.610359 |
|  | 449.315 | 1.13763 | 312.357 | 1.23334 |
|  | 686.992 | 0.940978 | 281.314 | 0.843174 |
|  | 495.529 | 1.07709 | 302.649 | 1.08992 |
|  | 402.276 | 0.769864 | 283.168 | 0.516657 |
|  | 392.364 | 1.067 | 299.769 | 0.996078 |
|  | 363.865 | 0.804672 | 328.191 | 0.772675 |
|  | 275.936 | 0.99775 | 346.894 | 0.939691 |
|  |  |  |  |  |
| **Mean** | 454.195 | 0.940644 | 312.3761 | 0.875237 |
| **Std. Error** | 45.34985 | 0.05487 | 9.019643 | 0.084871 |
| **n** | 8 | 8 | 8 | 8 |
| **t-test** |  |  | 0.024542 | 0.112045 |

**Figure 2:**

|  | **IPSC (AM251)** | | | |
| --- | --- | --- | --- | --- |
|  | **control** | | **CB65** | |
|  | **amplitude** | **PPR** | **amplitude** | **PPR** |
|  | -484.874846 | 0.977299 | -201.941 | 0.816123 |
|  | -441.81292 | 0.814297 | -268.712 | 0.821213 |
|  | -851.181 | 0.830352 | -268.712 | 0.747262 |
|  | -505.71436 | 0.940036 | -241.777 | 0.933947 |
|  | -252.514567 | 0.757931 | -308.365 | 0.683012 |
|  | -572.477533 | 0.88761 | -498.88 | 0.85481 |
|  | -1624.72617 | 0.86798 | -1105.89 | 0.783441 |
|  | -479.689567 | 0.86402 | -203.378 | 1.05775 |
|  |  |  |  |  |
| **Mean** | -651.62387 | 0.867440625 | -387.206 | 0.837195 |
| **Std. Error** | 150.8890538 | 0.024622114 | 107.9462 | 0.040919 |
| **n** | 8 | 8 | 8 | 8 |
| **t-test** |  |  | 0.009561 | 0.441598 |

**Figure 3:**

|  | **DSI** | | | | | | | | |
| --- | --- | --- | --- | --- | --- | --- | --- | --- | --- |
|  | **control** | | **AM251** | |  | **control** | | **SR144528** | |
|  | **Baseline** | **DSI** | **Baseline** | **DSI** |  | **Baseline** | **DSI** | **Baseline** | **DSI** |
|  | -296.81 | -234.78 | -477.00 | -462.68 |  | -1004.06 | -486.31 | -340.23 | -163.69 |
|  | -1023.24 | -612.49 | -2273.90 | -2483.45 |  | -829.90 | -330.22 | -378.64 | -207.57 |
|  | -888.76 | -335.63 | -1528.12 | -1501.10 |  | -877.74 | -700.91 | -1140.29 | -704.16 |
|  | -962.89 | -553.07 | -400.34 | -388.87 |  | -918.41 | -464.08 | -819.49 | -664.60 |
|  | -745.37 | -372.86 | -243.16 | -212.11 |  | -558.66 | -247.39 | -482.83 | -222.32 |
|  | -321.63 | -212.33 | -217.85 | -154.05 |  | -521.57 | -370.14 | -967.56 | -528.53 |
|  | -374.13 | -276.11 | -312.48 | -368.70 |  | -664.46 | -440.77 | -366.67 | -228.61 |
|  | -501.76 | -260.76 | -275.68 | -346.31 |  | -830.61 | -512.33 | -1698.52 | -782.23 |
|  |  |  |  |  |  |  |  |  |  |
| **Mean** | -639.32 | -357.25 | -716.07 | -739.66 |  | -775.68 | -444.02 | -774.28 | -437.71 |
| **Std. Error** | 106.32 | 52.80 | 269.65 | 290.75 |  | 61.67 | 48.17 | 170.27 | 91.38 |
| **n** | 8.00 | 8.00 | 8.00 | 8.00 |  | 8.00 | 8.00 | 8.00 | 8.00 |

**Figure 4:**

|  | **IPSC (GDP-β-S)** | | |
| --- | --- | --- | --- |
|  | **control** | **CB65** |  |
|  | -826.974591 | -532.234329 |  |
|  | -575.523332 | -768.162508 |  |
|  | -306.957032 | -267.776818 |  |
|  | -265.850731 | -219.623506 |  |
|  | -784.210143 | -522.430738 |  |
|  | -503.946864 | -257.18482 |  |
|  | -585.561876 | -560.942944 |  |
|  | -664.799917 | -730.955525 |  |
|  |  |  |  |
| **Mean** | -564.228061 | -482.413898 |  |
| **Std. Error** | 71.5861749 | 75.53753158 |  |
| **n** | 8 | 8 |  |
| **t-test** |  | 0.221863358 |  |

|  | **RuBi-GABA** | |
| --- | --- | --- |
|  | **control** | **CB65** |
|  | -574.331 | -301.834 |
|  | -819.13 | -287.288 |
|  | -1145.94 | -561.396 |
|  | -368.663 | -169.74 |
|  | -271.255 | -215.854 |
|  | -346.97 | -321.317 |
|  |  |  |
| **Mean** | -587.715 | -309.571 |
| **Std. Error** | 138.014 | 55.51149 |
| **n** | 6 | 6 |
| **t-test** |  | 0.034194 |
